# Trust and the communication of flood risks: comparing the roles of local governments, volunteers in emergency services, and neighbours

**DOI:** 10.1111/jfr3.12313

**Published:** 2017-07-31

**Authors:** S. Seebauer, P. Babcicky

**Affiliations:** ^1^ Joanneum Research Forschungsgesellschaft mbh LIFE – Centre for Climate, Energy and Society Graz Austria; ^2^ Wegener Center for Climate and Global Change University of Graz Graz Austria; ^3^ FWF‐DK Climate Change University of Graz Graz Austria

**Keywords:** Climate change adaptation, information source, private flood preparedness, risk communication, trust

## Abstract

Risk information need to be communicated by trusted groups, in order to promote attitude and behaviour change. We compare different levels of trust in local governments, volunteers in emergency and relief services, and neighbours, and how trust in these groups shapes citizens’ perceptions and actions relating to flood risks. Structural equation modelling is applied to a sample of 2007 flood‐prone households in Austria. A series of cognitive and behavioural responses to flood risks is regressed on trust shown to the three groups. Our findings show that citizens show great trust and attribute high competence to volunteers, which increases risk perception and reduces denial and wishful thinking. Trust in local government downplays risks, makes citizens rely on external help, and promotes fatalism and wishful thinking. Trust in neighbours increases reliance on social support and reinforces wishful thinking. These trust effects reflect the roles and risk narratives of the respective groups. To stimulate specific actions of citizens in flood risk management, the group which addresses the desired actions within its narrative should act as risk communicator. Risk communication could be introduced as a complementary activity in voluntary emergency and relief services, wherein older, retired volunteers seem particularly qualified as risk communicators.

## Introduction

Numerous Western countries are facing increasing flood risks in residential areas, caused by a lack of stringent regulation in settlement development, accumulation of economic assets in risk areas, and climate change (IPCC (Intergovernmental Panel on Climate Change), 2013; APCC (Austrian Panel on Climate Change), 2014). To keep future flood hazards manageable and to complement structural public flood protection, European and national flood policies emphasise the role of flood‐prone households in risk reduction (EU, 2007; Van Aalst *et al.,* 2008; BMLFUW (Ministry of Agriculture, Forestry, Environment and Water Management), 2014; Veraart *et al.,* 2014). Most recent risk mitigation strategies delegate the lead responsibility for managing local risks to local actors (Fuchs, 2009; Thaler and Levin‐Keitel, 2015). Risk managers, however, seem to struggle to develop effective risk communication strategies that build awareness and encourage residents in risk‐prone areas to take precautionary action (Kellens *et al.,* 2013).

Risk information is more likely to be adopted and translated into action if it is conveyed by trusted communicators (Renn and Levine, 1991; Kasperson, 1992; Slovic, 1993, 1999; Breakwell, 2000; Earle, 2010). Research on households at risk of wildfires, for instance, shows that messages from credible and trustworthy sources positively influence message elaboration and intention to take protective action (Bright *et al.,* 2006). Risk communicators originating from the same regional context and social milieu, and holding similar worldviews to the targeted citizens, can function as multipliers and change agents (McKenzie‐Mohr, 2000; Rogers, 2003). They are particularly qualified to influence citizens by means of social norms, professional knowledge, new social contacts or new ideas, eventually triggering preventive action. This paper compares how three stakeholder groups attract trust as risk communicators: local governments, volunteer workers in disaster emergency and relief services, and neighbours. We show that volunteers are the most trusted group and that they shape citizens’ perceptions and actions concerning flood risks.

The remainder of the paper is organised as follows: Section *Theoretical background* provides the theoretical background on different stakeholder groups who communicate flood risks, as well as on different facets of trust and cooperation. Section *Method* presents data, operationalization, and analytical procedures. In Section *Results and discussion*, we show that volunteers are the most trusted group, and reveal group‐specific impacts of trust on citizens’ risk perception, intentions for implementing private flood mitigation, and self‐justifications for refraining from taking action (e.g. denial or wishful thinking). Section *Conclusions and policy implications* concludes.

## Theoretical background

### Stakeholder groups in risk communication

Trust evolves from social relations, in our case when citizens (the trustors) place trust in various stakeholder groups (the trustees). Previous studies show that citizens demonstrate trust differently towards public authorities, scientific experts, media, industry representatives, and other actors (Frewer *et al.,* 1996; Bright *et al.,* 2006; Harvey and Twyman, 2007; Earle, 2010). Most of these studies, however, address modern technologies, such as genetically modified organisms or nuclear waste. Only a few studies explore how trust varies across different stakeholder groups in the context of natural hazards (e.g. Haynes *et al.,* 2008; Eiser *et al.,* 2015).

In flood risk management, three trustees take a central role in risk communication: First, the local government, which has the statutory obligation to inform about natural hazards and protect its citizens from them. Secondly, neighbours, who can be considered a household's most proximal reference regarding disaster risks, as they live in the same regional context and jointly contribute to the social resilience of the community (Tierney, 2014). Thirdly, volunteers in emergency and relief services (such as fire brigades or ambulance services), which are an integral part of organised flood responses in numerous countries.

Voluntary emergency and relief services form the backbone of disaster risk management throughout the world (Alexander, 2002). In Austria, the geographical focus of this study, 5% of all citizens volunteer in emergency and relief services, providing a total of 1.4 million service hours per week (BMASK (Ministry of Labour, Social Affairs and Consumer Protection), 2013). Volunteers contribute the main workforce of trained, rapidly deployable, and localised personnel, if a major disaster strikes in Austria (BMI (Ministry of the Interior), 2009; BMLFUW (Ministry of Agriculture, Forestry, Environment and Water Management), 2012). Most volunteers are tightly interwoven into the social fabric of their communities (Pelling and High, 2005; Kuhlicke *et al.,* 2011; Balas *et al.,* 2015) and generate numerous societal benefits, by fostering social capital and relieving public budgets (Bachner *et al.,* 2016). Volunteers also act as local mediators between experts and the local community, bridging the gap between the different knowledge domains of the actors involved in disaster risk management (Begg *et al.,* 2012). This makes them a vital link in local (risk) communication networks. While the majority of volunteer organisations traditionally focuses on the direct response to disaster events, this paper examines whether volunteers could expand their current activities to risk prevention and risk communication. Since volunteers are recruited from various societal groups and have rich practical experiences with flood events, they seem particularly qualified to communicate risks in flood‐prone communities.

Neighbours exert social influence on flood‐prone households to engage in flood mitigation activities (Kunreuther and Michel‐Kerjan, 2009). By conveying social expectations of what a household at risk should do, and by carrying the risk debate into informal personal networks, neighbours disseminate risk information and contribute to the social amplification of risk (Kasperson *et al.,* 1988). Households observe the actions of their neighbours and tend to follow suit in their decisions about how much to spend on flood mitigation (Lo, 2013). This peer influence operates across a number of different mitigation activities, such as the use of flood insurance, flood warnings, and floodgates (Dittrich *et al.,* 2016). Neighbours in disaster‐prone areas often pass on warnings to other households, provide emergency assistance (Werritty *et al.,* 2007) or introduce new community members to the risks of their environment (Wood *et al.,* 2013). These examples provide evidence of the active role of neighbours in risk communication. However, detailed insights on how social interactions between households and their neighbours unfold (Renn, 2011) are still lacking.

### The role of trust in risk communication

Trust comprises (1) a social‐relational aspect between trustor and trustee and (2) a calculative assessment of the trustee's competence by the trustor (Earle, 2010). The first dimension refers to the trustor's appraisal of the trustee's intentions, for example whether citizens at risk from flooding believe that local authorities would do everything in their power to protect them from harm. This dimension also reflects procedural fairness, like transparent and inclusive communication and decision‐making. The second dimension of trust comprises the trustor's assessment of the trustee's abilities and expertise in understanding and coping with past and future risks. In the flood example, this would be how citizens judge the capabilities of authorities in designing and implementing appropriate protective measures.

The Trust‐Confidence‐Cooperation Model by Earle et al. (2007) proposes different antecedents for these two dimensions. According to this model, value similarity precedes trust, and past performance precedes competence. Similar values make it more predictable how trustees intend to act, thus forming the basis for trust (Earle, 2010; Siegrist, 2010). Past performance signals‐specific abilities applied by the trustees in past situations, thus substantiating expectations of their future capabilities (Siegrist *et al.,* 2012).

However, trust, competence, and past performance often cannot be confirmed as conceptually distinct factors (Earle, 2010; Siegrist, 2010). This ambiguity further blurs in the general concept of trustworthiness as an overall opinion on the ability, benevolence, moral integrity, honesty, concern and accountability of a trustee, above and beyond a specific hazard (Sharp *et al.,* 2013). In the context of flood risk, it seems likely that trust and competence converge into a joint factor, due to the rare recurrence of flood events: As many people personally experience a flood just once in their lifetime (or not at all), they often lack first‐hand knowledge of this hazard or of a risk manager's operational performance. Instead, they fall back on trust as a decision heuristic when making judgments (Siegrist, 2010; Kellens *et al.,* 2013).

Despite this conceptual ambiguity, trust is found to be more relevant for effective risk communication than that for competence and past performance (Edwards and Cable, 2009; Earle, 2010). Trust is more resilient to change, because it is connected with various situations and expectations of how it should be employed; trust enables acceptance of unfamiliar and uncontrollable risks by transferring the responsibility to deal with uncertain outcomes from the trustor to the trustee (Siegrist, 2000; Kellens *et al.,* 2013; Sharp *et al.,* 2013).

Trust influences various forms of cooperation: risk perception (Siegrist, 2000; Siegrist and Cvetkovich, 2000; Siegrist *et al.,* 2005; Earle, 2010; Lu *et al.,* 2015) and negative emotions associated with risks (like fear, worry, or concern; Kellens *et al.,* 2013), as well as information‐seeking and adaptive behaviour such as private flood mitigation (Earle, 2010; Terpstra, 2011; Kellens *et al.,* 2013). Furthermore, trust in various stakeholders affects the perception of more general risks, such as climate change (Kellstedt *et al.,* 2008; Malka *et al.,* 2009). The Trust‐Confidence‐Cooperation Model proposes a broad understanding of ‘cooperation’ – cooperative responses include protective behaviours as well as antecedents or supporting/hindering factors of these very behaviours. In that sense, risk perception is also a form of cooperation, as it is considered a key antecedent of protective action (Van der Pligt, 1996). Thus, following the broad understanding of cooperation, this study illustrates the impact of trust on the perception of proximal risks (e.g. a flood affecting one's own residence) and distant ones (e.g. climate change as a driver of future flood risks).

Not all citizens naturally respond to receiving risk information by implementing private flood mitigation measures. Instead, some turn to non‐protective responses to suppress the negative emotional consequences associated with a certain risk (Milne *et al.,* 2000; Grothmann and Reusswig, 2006; Bubeck *et al.,* 2013). Non‐protective responses, such as denial, wishful thinking and fatalism, are used to justify inaction, and therefore undermine an individual's protection motivation. Non‐protective responses are shaped by risk perception, but they are conceptually distinct, since they are reactions to risks that exceed an individual's coping capacities. For risk communication to be effective, it appears critical to develop a more precise understanding of why some households translate information into action and others do not. We therefore investigate protective responses as a form of cooperation, as well as non‐protective responses as a form of non‐cooperation.

As discussed above, citizens show trust differently to various trustees. Not only does the extent of trust in them vary between trustees, but so does the strength of the relationship between trust and certain forms of cooperation (Earle, 2010). In the case of ‘information on food safety’, Lobb *et al.* (2007) show that trust in media increases risk perception and reduces the likelihood of purchasing groceries, while trust in public authorities has opposite effects on risk perception and purchase intentions. Thus, the impact of trust on cooperation may depend on the respective trustee.

Levels of trust are also influenced by characteristics of the trustor. Having been personally affected by a flood or living in a flood risk zone are key factors in individual risk assessment (Terpstra, 2011; Bubeck *et al.,* 2013; Terpstra and Lindell, 2013); thus, these factors may also determine levels of trust in flood risk communication. In the context of nutritional risks, previous experience and perceived susceptibility influence trust in institutions (Kuttschreuter, 2006).

Overall, as regards our case of citizens (the trustors) demonstrating trust in various stakeholder groups in flood risk communication (the trustees), existing literature indicates that (1) different trustees receive different levels of trust and (2) trustees have different impacts on certain forms of cooperation by their trustors. This study sets out to empirically test these assumptions in the context of flood risks in Austria by systematically comparing the following trustees: local government, volunteers, and neighbours.

## Method

### Study setting and data collection

This cross‐sectional study is based on a postal questionnaire survey, administered between October 2014 and February 2015, in 10 municipalities in Austria. The municipalities are primarily situated in Alpine mountain regions and had either been recently affected by a flood event, or are at risk of severe riverine flooding or pluvial torrents. Most of the surveyed municipalities are small rural communities of 500–3000 households, except Lustenau, which has a population of 8000 households. The questionnaires were distributed as an insert to municipal newspapers; these newspapers were delivered by post to all residents in the surveyed municipalities. The questionnaires could be returned in a dedicated freepost return envelope, dropped off at the local municipal office, or completed in an identical online survey. Respondents had the offer of participation in a lottery for gift vouchers upon completing the survey (5 × 30 Euro in each municipality). Local authorities also promoted the survey through their civic communication channels. Overall, 2007 valid questionnaires were returned, resulting in a response rate of 12.8%. Respondents from the municipality of Lustenau form the majority of the sample (Table S1, Supporting Information); yet, the results are robust and apply to the Lustenau subsample in the same way as to the subsample of the other municipalities.

Socio‐demographics in the sample conform fairly well to census data of the residential population in the surveyed municipalities (Table S2). Male, older, and lower‐income respondents are to some degree overrepresented in the sample. Precise risk zone data at a municipal level are not available in Austria; the sample statistics, however, indicate that households at risk (having previously experienced a flood at their residence or living in a risk zone) were more willing to participate in the survey than households that are not at risk. However, checks on the robustness of the results do not indicate bias due to restricted representativeness.

### Measures

All concepts are specified as latent factors, measured on continuous multi‐item scales, in order to correct for measurement error of single items. Detailed item wordings, descriptive statistics and factor loadings are given in Tables S3 and S4.

Unless stated differently, all items are rated on a Likert‐type bipolar response scale, ranging from 1 = fully disagree to 5 = fully agree. All original items were in German and have been translated for this paper. In the questionnaire, items were presented in mixed order, so that their conceptual assignment to factors was not transparent to the respondents. When referring to specific trustees, the local government was introduced as the mayor and elected representatives in the municipal council; volunteers referred to volunteers in disaster emergency and relief services, such as fire brigades, ambulances, or mountain rescue services.

#### Trust, competence, and past performance

Trust, competence, and past performance are measured separately for local government, volunteers, and neighbours. Three trust, two competence, and two past performance items are derived from previous studies on trust and credibility of stakeholder groups (Frewer *et al.,* 1996; Siegrist, 2000; Earle and Siegrist, 2006; Terpstra *et al.,* 2014).

#### Value similarity

Respondents indicated how similar they perceived the values of the respective trustees to be to the values they held themselves. Value similarity is assessed by means of three items adapted from empirical studies based on the Trust‐Confidence‐Cooperation model (Siegrist *et al.,* 2000; Earle and Siegrist, 2006).

#### Risk perception

Perceived flood risk is measured separately for the entire home municipality and for the respondent's own building. Flood risk perception items assess the perceived probability and severity of a severe local flood event within the next 10 years (adapted from Terpstra and Lindell, 2013). Further items capture fear associated with flood risks (two items), and risk perception of climate change (three items).

#### Intention of implementing private measures

Respondents were asked to assess seven protective measures, ranging from preparing sandbags to retrofitting existing buildings. Intention of implementing each measure was rated on a six‐step rating scale from already implemented (6), very likely (5), rather likely (4), rather unlikely (3), very unlikely (2), and to not feasible (1). Within each household, all seven measures are aggregated to a formative mean index, in order to reflect the prospect that a household will implement a set of combined, interlinked mitigation measures. Note that this index does not suggest whether the implementation of a single measure might be enough to achieve the desired level of protection, or whether a household was prepared or not; both of these aspects would have required an *in situ* assessment of each residential building.

#### Fatalism, denial, and wishful thinking

Non‐protective responses include fatalism, denial, and wishful thinking and are captured by four, three, and four items, respectively. Item wordings are taken from Grothmann (2005).

#### Reliance on public protection, reliance on social support

Two additional aspects of non‐protective responses are included to account for further reasons to refrain from protective action. Reliance on public protection relates to the feeling of being protected from flooding by public authorities and is found to negatively affect mitigation behaviour (four items; Grothmann and Reusswig, 2006; Poussin *et al.,* 2014). Reliance on social support reduces a household's flood risk perception and may thus undermine protection motivation (three items; Babcicky and Seebauer, 2017).

#### Socio‐demographic characteristics

Besides stating gender, age, and income, respondents indicated whether they had been previously affected by a flood. Whether respondents live in a flood risk zone or not is assessed according to the classification scheme used in Austria's official flood risk zone database (HORA, 2015).

### Data analysis procedure

We employ a two‐step approach to analysing the survey data. First, we test for mean differences in the levels of trust, competence, past performance, and value similarity between trustees. We apply one‐way analyses of variance for repeated measures, as each respondent assessed all three trustees. We then show whether households display trust differently if they are exposed to flood hazards. For the sake of brevity, Section *Levels of trust, competence, past performance, and value similarity* only gives the mean values within each compared group, whereas full test statistics are reported in the Appendix S1.

Second, we estimate a series of 10 structural equation models for 10 different forms of cooperation, within each model determining the unique impact of each trustee while controlling for the impacts of the other trustees. We use raw input data and apply the full information maximum likelihood procedure for estimating our models while accounting for missing values. All models are computed with AMOS 21 software. Model fit indices reveal how well the proposed models reflect the observed data. A good model fit is indicated by a comparative fit index (CFI) and normed fit index (NFI) larger than 0.90 and close to 1, a root mean‐square error of approximation (RMSEA) lower than 0.08, and a ratio of Chi^2^ to degrees of freedom (df) lower than 5 (Schumacker and Lomax, 2004). The Chi^2^ statistic also serves as a criterion in model comparisons, if restricted models (assuming equal impacts of all trustees) fit significantly better or worse to the observed data.

In both analytical steps, the key variables are the various dimensions of trust shown to the respective trustees. To confirm that these dimensions are captured with valid measures, we conduct a separate confirmatory factor analysis (CFA) of the trust, competence, past performance, and value similarity factors. A full account of the CFA rationale and results are available in the Appendix S1. The measurement validity of the other factors, which represent various forms of cooperation, is reflected in the general model fit.

The CFA results confirm that the three trustees are perceived differently; in other words, that the assessments of local government, volunteers, and neighbours do not converge into a general assessment encompassing all trustees. However, the CFA results also imply that citizens have an overarching concept of the trust, competence, and past performance they ascribe to a specific trustee. This overlap between these trust dimensions impairs the statistical measurement properties required for structural equation models.

We therefore use mean indices instead of latent constructs for our first analytical step of mean comparisons. The second step, contrasting trustee‐specific impacts on cooperation, focuses solely on the trust dimension. By doing this, we avoid conceptual ambiguity and account for the central role of trust for effective risk communication (see Section *The role of trust in risk communication*). We retain value similarity as a separate concept, since it can be clearly distinguished from trust.

## Results and discussion

### Levels of trust, competence, past performance, and value similarity

The absolute scores for trust, competence, past performance, and value similarity are generally high, with mean values of the respective indices scoring in the positive spectrum of the five‐step rating scale. Our results show that volunteers score higher on all four factors than the local government and neighbours (Table 1; for detailed test statistics, see Tables S8–S10). The central role of volunteers in coping with disasters and their strong integration into local communities are probably among the reasons why this group enjoys higher trust, competence, past performance, and shared values than the two other trustees. The local government scores higher than neighbours in terms of competence and past performance, but these mean differences are small in absolute terms and yield statistical significance primarily due to the large sample size.

**Table 1:** Mean levels of trust, competence, past performance, and value similarity indices by trustee, flood experience and risk zone

		Local government		Volunteers		Neighbours	
Trust	All	3.75		4.39		3.64	**
	No flood experience	3.84	##	4.39		3.61	
	Flood experience	3.50		4.39		3.69	
	Not in risk zone	4.09	##	4.60	##	3.90	##
	In risk zone	3.61		4.38		4.35	
	Does not know risk zone	3.73		4.35		3.56	
Competence	All	3.59		4.14		3.16	**
	No flood experience	3.69	##	4.16		3.15	
	Flood experience	3.33		4.14		3.20	
	Not in risk zone	3.93	##	4.45	##	3.55	##
	In risk zone	3.44		4.10		3.14	
	Does not know risk zone	3.58		4.10		3.08	
Past performance	All	3.72		4.30		3.38	**
	No flood experience	3.84	##	4.30		3.33	##
	Flood experience	3.44		4.32		3.51	
	Not in risk zone	4.11	##	4.56	##	3.74	##
	In risk zone	3.51		4.25		3.38	
	Does not know risk zone	3.71		4.26		3.31	
Value similarity	All	3.90		4.31		4.02	**
	No flood experience	3.96	##	4.30		3.96	##
	Flood experience	3.74		4.35		4.19	
	Not in risk zone	4.09	#	4.41	#	4.11	
	In risk zone	3.88		4.39		4.12	
	Does not know risk zone	3.85		4.27		3.95	

Results obtained by analyses of variance read as following: Rows indicated with “all” refer to the full sample and mean values are compared *between* trustees, i.e. row‐wise comparison (*P < 0.0125, **P < 0.0025). For comparisons by “flood experience” and “risk zone”, mean values are compared *within* each trustee, i.e. column‐wise comparison (#*P* < 0.001, ##*P* < 0.0002). All significance levels are Bonferroni adjusted to control for cumulative type 1 error. For standard deviations and test statistics, see Tables S8‐S10.

Mean levels of trust, competence, past performance, and value similarity tend to be higher among trustors who have not been personally affected by a flood or do not live in a flood risk zone (Table 1). Households without previous flood experience assess the local government by up to 0.4 steps higher on all four trust factors than households with flood experience. By contrast, having experienced a flood implies a more positive evaluation of neighbours’ past performance and value similarity; yet, this discrepancy amounts to no more than 0.2 scale steps. Households living outside a flood risk zone rate trust, competence and past performance for all trustees by up to 0.6 steps higher than households living in a risk zone; households that do not know whether their residential location is at risk widely conform with the assessments of those in risk zones.

Those mean differences suggest that households show more trust and attribute more competence, past performance, and value similarity to a trustee, if they are not exposed to flood hazards. *Vice versa*, if households are directly threatened by a flood, they seem to be more reserved in their attributions, as they might have realised the limits of the trustees’ coping capacities.

### Trustee‐specific impacts on cooperation

The key objective of this paper is to determine whether trust in specific trustees has different impacts on citizens’ perceptions and actions. To this end, various forms of cooperation are regressed on trust shown to the local government, volunteers, or neighbours, respectively.

**Figure 1:**
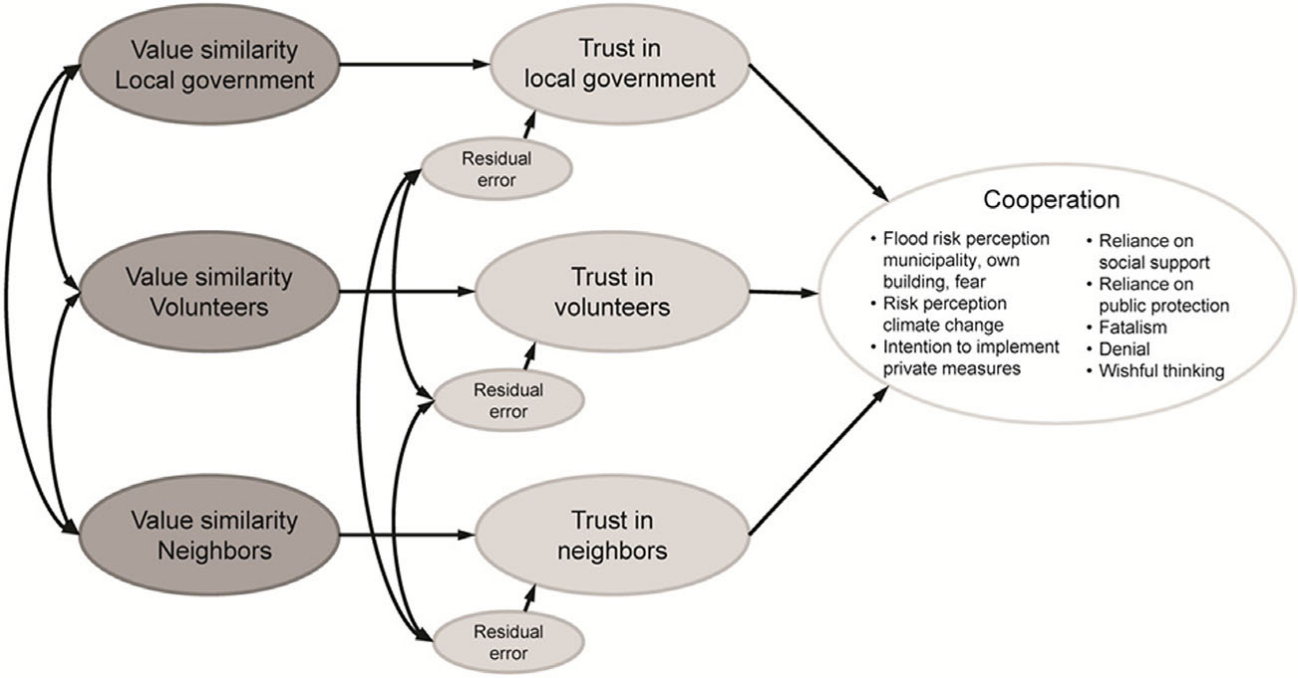
Structural model for trustee‐specific impacts on cooperation.

As introduced in Section *The role of trust in risk communication*, our models include value similarity as an antecedent of trust. The trustee‐specific value similarity factors and trust factors are intercorrelated to account for social interrelations and shared beliefs within a community (for intercorrelation coefficients, see Table S6). Figure 1 summarises the structural model, as it is applied to all 10 forms of cooperation shown in Tables 2 and 3; each column in these tables refers to a separate model. The CFI, NFI, and RMSEA indices point to an excellent fit in all models.

**Table 2:** Trustee‐specific impacts on risk perception

	**Flood risk perception (municipality)**	**Flood risk perception (own building)**	**Flood risk perception (fear)**	**Risk perception (climate change)**
Trust in local government	−0.41 **	−0.33 **	−0.22 **	0.02
Trust in volunteers	0.16 **	0.09 **	0.03	0.02
Trust in neighbours	0.00	−0.01	0.04	0.01
*R* ^2^	12.6%	8.8%	3.7%	0.0%
Chi^2^ (df)	1293 (162) **	1294 (162) **	1346 (162) **	1311 (181) **
CFI	0.96	0.96	0.96	0.96
NFI	0.95	0.96	0.95	0.96
RMSEA (10% CI)	0.059 (0.056–0.062)	0.059 (0.056–0.062)	0.060 (0.057–0.063)	0.056 (0.053–0.059)
Model comparison to equal impacts of trustees Difference in Chi^2^ (df)	103 (2) **	63 (2) **	33 (2) **	0.1 (2)

CI, confidence interval

* *P* < 0.05,

** *P* < 0.01;

standardised path coefficients.

**Table 3:** Trustee‐specific impacts on private action and non‐action

	**Intention of implementing private measures**	**Reliance on social support**	**Reliance on public protection**	**Fatalism**	**Denial**	**Wishful thinking**
Trust in local government	−0.06 *	0.20 **	0.81 **	0.27 **	0.19 **	0.37 **
Trust in volunteers	0.09 **	0.13 **	0.00	0.03	−0.13 **	−0.10 **
Trust in neighbours	0.13 **	0.31 **	−0.04	0.00	0.05	0.10 **
*R* ^2^	2.7%	26.0%	63.0%	8.4%	3.1%	13.7%
Chi^2^ (df)	1236 (145) **	1330 (181) **	1500 (201) **	1401 (201) **	1303 (181) **	1389 (201) **
CFI	0.96	0.96	0.96	0.96	0.96	0.96
NFI	0.96	0.96	0.95	0.95	0.96	0.95
RMSEA (10% CI)	0.061 (0.058–0.064)	0.056 (0.053–0.059)	0.057 (0.054–0.059)	0.055 (0.052–0.057)	0.056 (0.053–0.058)	0.054 (0.052–0.057)
Model comparison to equal impacts of trustees Difference in Chi^2^ (df)	21 (2) **	11 (2) **	540 (2) **	26 (2) **	29 (2) **	49 (2) **

CI, confidence interval

* *P* < 0.05,

** *P* < 0.01;

standardised path coefficients.

The impact of value similarity on trust, that is to say, the left‐hand part of the structural model, is identical for all 10 models. More similar values lead to higher levels of trust for all three trustees. This effect is most evident for the local government, with *β* = 0.75. Regarding volunteers and neighbours, value similarity influences trust with *β* = 0.56 and 0.53, respectively.

The path coefficients in Tables 2 and 3 confirm that trustee‐specific trust indeed has differential impacts: (1) when comparing the local government, volunteers, and neighbours within the same form of cooperation (column‐wise in Tables 2 and 3) and (2) when comparing forms of cooperation within the same trustee (row‐wise in Tables 2 and 3). Model comparison establishes statistical significance for the (1) column‐wise differences. Within each form of cooperation, we compare the reported model to a restricted model, wherein the coefficients of the three trustee‐specific trust paths are set to be equal. Thus, the restricted model assumes that trust exerts equal influence on cooperation, regardless of trustee. The bottom row in Tables 2 and 3 shows that for all forms of cooperation, the restricted model fits significantly worse to the data than the model allowing for different impacts by trustee – except for risk perception of climate change, where trust impacts are so weak that they do not differ between trustees.

The amount of explained variance (*R* ^2^) reveals how strongly the various forms of cooperation depend on trust (Tables 2 and 3). Flood risk perception in the municipality, wishful thinking, reliance on social support, and, most of all, reliance on public flood protection are heavily influenced by trust. Contrastingly, flood risk perception for their own building, fatalism, fear associated with flood risk, denial, and intention of implementing private measures are less strongly determined by trust. Risk perception of climate change is virtually unrelated to trust; thus, the impact of trust does not seem to extend to an underlying driver of flood risks. Low *R* ^2^ values suggest that these forms of cooperation depend on other drivers not being included as predictors, such as threat appraisal, coping appraisal, or other adaptive capacities.

Our main focus, however, is to compare the impacts of the three trustees. The patterns of influence on cooperation as shown in Tables 2 and 3 reflect the trustees’ roles and narratives within disaster management. The more households trust the local government, the less they perceive themselves to be at risk from flooding (*γ* = −0.22 to −0.41). Higher trust in the local government also makes households less willing to implement private measures (*γ* = −0.06), makes them rely more on external help to cope with a flood event (*γ* = 0.20 and 0.81), and makes them revert more strongly to non‐protective responses (*γ* = 0.19 to 0.37). Presumably, these effects mirror the way that risk communication is framed by local governments – trivialising potential risks, assuring citizens that they are sufficiently protected by public measures, and emphasising that the consequences of a flood can be controlled effectively.

In contrast, trust in volunteers leads to higher flood risk perception (*γ* = 0.16 and 0.09), higher intention of implementing private measures (*γ* = 0.09), higher reliance on social support (*γ* = 0.13) and reduces denial and wishful thinking (*γ* = −0.13 and −0.10, respectively). Possibly, volunteers communicate from the viewpoint of how they have experienced disaster emergency operations themselves, emphasising that disasters often require more coping capacities than initially expected or cause more severe damages than a community was prepared for. Drawing on their hands‐on experience with actual flood events, volunteers may promote a more realistic risk assessment among households at risk. Being the main workforce in disaster response and recovery, volunteers are an important source of social support to flood‐affected households.

High trust in neighbours makes citizens more likely to intend to implement private measures (*γ* = 0.13), rely heavily on social support (*γ* = 0.31), and to indulge in wishful thinking (*γ* = 0.10). Since neighbours often share similar physical risks on a property‐level scale, they may be particularly qualified to credibly address and promote actions that can be taken by a household to improve its resilience to flooding. Alternatively, trust in neighbours might be a proxy for social norms, wherein neighbours function as role models in private flood preparedness. However, despite their geographical and social proximity, the influence of neighbours does not appear to extend to the formation of individual risk assessments, as risk perception is virtually unrelated to trust in neighbours (*γ* = −0.01 to 0.04).

Overall, the model comparison reveals that the three trustees have different impacts on cooperation and thus, presumably, communicate different forms of cooperation. Depending on the type of cooperation risk managers aim to address, they may leverage the proper trustee as a change agent. Among the three trustees, volunteers are the most qualified multipliers to promote flood risk awareness and to reduce the adoption of non‐protective responses, such as denial and wishful thinking, among citizens.

## Conclusions and policy implications

This paper examines how households show trust in various stakeholder groups in flood risk communication and how trust in these groups influences risk perception and (non‐)protective behaviour. We show that trust in the local governments, in volunteers in disaster emergency and relief services, and in neighbours affects how citizens perceive and act on flood risks. Among these three groups of trustees, we identify volunteers as specifically qualified to build awareness and communicate risks: Volunteers are the most trusted group and are perceived as more competent in the field of flood risk mitigation than the other two groups. Trust in volunteers increases risk perception and reduces the adoption of non‐protective responses such as denial and wishful thinking.

Our results reflect that the narratives of stakeholder groups in the field of risk communication trigger different forms of civic cooperation. Tierney (2014) argues that the way a society identifies and is concerned about risks is an outcome of narratives produced by societal actors. It appears that volunteers address specific elements relevant to sparking private action and cooperation in flood risk mitigation, which are not covered in the risk narratives put forward by local governments or neighbours. Thus, volunteers could be a more effective communication channel in flood risk communication than other stakeholders.

Even though most voluntary emergency and relief services currently conduct risk communication only as a complementary activity, they can profit from promoting preventive action in the long term. Flood risks are expected to increase as a consequence of socio‐economic change, spatial development, and climate change. If flood‐prone households engage in preventive action, they would require less external support from volunteers when responding to and recovering from a flood event. This would free up resources and make it more likely that the operational capacities of the current volunteer system might suffice to handle increasing risks.

Voluntary organisations are firmly entrenched in the social structure of Austrian communities. This puts them in a key position to advance citizens’ awareness of flood risks, which may then pave the way for private flood adaptation as well as a greater acceptance of public measures, such as built infrastructure or mandatory flood insurance. However, it is crucial to note that the socially disadvantaged are generally less likely to engage in voluntary work (Wilson and Musick, 1998), and the role of volunteer risk communicators might therefore be restricted to better‐off social strata. Still, we recommend establishing risk communication as a regular activity of voluntary emergency and relief services, on top of their current core activities (Balas *et al.,* 2015).

Older, retired volunteers appear to be particularly qualified as risk communicators: They have extensive experience in flood emergency operations, command strong community resources and have numerous, historically grown social ties. Moreover, they are less able or willing to engage in active field operations than younger volunteers (BMASK (Ministry of Labour, Social Affairs and Consumer Protection), 2013). Taking responsibility for non‐emergency tasks might ease their transition from active duty to other, less physically demanding activities. On the other hand, volunteers would require special training in lay communication and technical knowledge about natural hazard prevention, and also in translating risk messages into the worldviews and decision heuristics prevalent in different social milieus.

Some voluntary organisations have already begun to deploy non‐emergency personnel. The initiatives *60PLUS* (Fire Brigade Vorarlberg, Austria)^1^ and *65plus* (Fire Brigade Baden‐Württemberg, Germany)^2^ aim to develop niches for elderly volunteers within existing organisational structures. The US *Fire Corps* 3 involves community members in support tasks like maintenance, reporting, and accounting to relieve qualified emergency personnel for operational duties. Insights from these initiatives can inform efforts to establish risk communication activities complementary to standard emergency and relief operations.

Future risk management would also benefit from including neighbours as risk communicators. While volunteers could spread risk messages on a wider scale, households are particularly qualified for peer‐to‐peer impulses directed towards private preparedness activities (McKenzie‐Mohr, 2000). Neighbourhood groups for flood resilience could share practical knowledge, jointly implement protection measures, or conduct emergency training. However, excessive reliance on social support, a dominant element in the risk narrative of neighbours, may spark a false sense of security that keeps households from taking protective action (Babcicky and Seebauer, 2017). While risk managers should welcome the use of neighbours to strengthen social arrangements for flood protection (Tierney, 2014), they also need to emphasise that neighbourly help is only effective as far as larger‐scale flood events do not exceed the available capacities for social support.

To date, flood risk communication in Austria has largely been in the hands of local governments. Their narratives appear to have made citizens perceive themselves as at lower risk, to have rendered them more prone to non‐protective responses, including fatalism, denial, and wishful thinking, and to relying heavily on public flood protection. Austrian local policy‐makers may be tempted to promise their voters that public efforts will contain impending flood risk. While these narratives seem unproblematic for municipalities in which adequate protective measures are already in place, they are counter‐productive for communities with insufficient protection. Communication practices that make residents feel safe, despite being at risk, need to be critically revised. One option for local governments might be to rethink how their messages are framed within the public discourse, and to include voluntary emergency and relief services strategically in their communication policies. Another option is to engage in trustful, bidirectional exchange with the public that results in mutual learning and in risk narratives compatible with the community's actual situation (Kellens *et al.,* 2013; Sharp *et al.,* 2013).

Our findings open new avenues for future research on the emergence of narratives in flood risk management. We argue that each trusted group conveys a specific risk narrative that promotes different forms of cooperation. However, the way these narratives evolve in intuitive, heuristic‐driven or elaborate, reflective processes, remains a black box that calls for a more detailed analysis. Finally, we encourage future studies to apply our approach of determining trustee‐specific narratives to other hazard contexts.

## Supporting information


**Appendix S1.**

## Footnotes

1
http://www.lfv-vorarlberg.at/verband/60plus.html

2
https://im.baden-wuerttemberg.de/fileadmin/redaktion/m-im/intern/dateien/publikationen/Feuerwehr-65plus_Broschuere.pdf

3
http://www.firecorps.org/
