# Supplementary material for: Trust and the communication of flood risks: comparing the roles of local governments, volunteers in emergency services, and neighbours
Source: J Flood Risk Manag. 2017 Jul 31;11(3):305–16. doi: 10.1111/jfr3.12313 (PMC6991925; doi:10.1111/jfr3.12313)
Supplement: Supplementary file 1 — Appendix S1. [file JFR3-11-305-s001.pdf]

Online Appendix

to

Seebauer, S., Babcicky, P.: Trust and the communication of flood risks: Comparing the roles of local governments, volunteers in emergency services and neighbors. *Journal of Flood Risk Management*.

1.1 Sample information

Table A.1: Sample distribution by municipality

Municipality									
Eisenerz	Fernitz	Gosdorf	Gössendorf	Hatzendorf	Mooskirchen	Radmer	Lustenau	Mellau	Nenzing
3.1%	2.2%	3.7%	7.6%	2.9%	2.5%	1.7%	65.0%	3.1%	8.2%

Table A.2: Sample and population socio-demographics

Municipality	Gender	Age (years)					Monthly net household income (€)						Flood experience	Risk zone		
	Female	20-34	35-49	50-64	65-79	80+	<1,100	1,100-1,599	1,600-2,599	2,600-3,999	4,000-5,500	5,501+	Yes	Yes	No	Don't know
Eisenerz SD	40%	9%	17%	31%	39%	5%	11%	30%	39%	11%	2%	7%	18%	25%	32%	43%
Eisenerz PD	53%	12%	18%	27%	29%	14%	7%	14%	21%	34%	17%	7%	n.a.	10-30%	n.a.	n.a.
Fernitz SD	25%	11%	46%	27%	16%	0%	10%	10%	17%	43%	17%	3%	63%	23%	8%	70%
Fernitz PD	52%	22%	30%	28%	14%	6%	7%	14%	21%	34%	17%	7%	n.a.	10-30%	n.a.	n.a.
Gosdorf SD	51%	6%	30%	36%	20%	7%	14%	32%	36%	16%	2%	0%	49%	28%	24%	48%
Gosdorf PD	50%	20%	28%	27%	18%	6%	7%	14%	21%	34%	17%	7%	n.a.	10-30%	n.a.	n.a.
Gössendorf SD	34%	13%	37%	32%	17%	1%	1%	17%	37%	32%	10%	4%	68%	44%	5%	52%
Gössendorf PD	51%	22%	32%	25%	15%	6%	7%	14%	21%	34%	17%	7%	n.a.	0-10%	n.a.	n.a.
Hatzendorf SD	29%	18%	32%	32%	18%	0%	13%	27%	31%	22%	4%	2%	50%	22%	29%	49%
Hatzendorf PD	50%	23%	28%	27%	16%	6%	7%	14%	21%	34%	17%	7%	n.a.	0-10%	n.a.	n.a.

Table A.2: Sample and population socio-demographics (cont.)

Municipality	Gender	Age (years)					Monthly net household income (€)						Flood experience	Risk zone		
	Female	20-34	35-49	50-64	65-79	80+	<1,100	1,100-1,599	1,600-2,599	2,600-3,999	4,000-5,500	5,501+	Yes	Yes	No	Don't know
Mooskirchen SD	54%	17%	28%	37%	17%	0%	13%	10%	43%	20%	13%	0%	48%	22%	33%	46%
Mooskirchen PD	51%	23%	29%	26%	14%	7%	7%	14%	21%	34%	17%	7%	n.a.	10-30%	n.a.	n.a.
Radmer SD	46%	6%	27%	36%	18%	12%	12%	54%	19%	12%	4%	0%	61%	42%	13%	45%
Radmer PD	50%	13%	22%	29%	23%	13%	7%	14%	21%	34%	17%	7%	n.a.	10-30%	n.a.	n.a.
Lustenau SD	34%	12%	25%	29%	26%	8%	6%	15%	38%	29%	9%	3%	15%	16%	9%	75%
Lustenau PD	51%	27%	29%	24%	16%	5%	8%	10%	36%	31%	8%	7%	n.a.	0-10%	n.a.	n.a.
Mellau SD	38%	8%	28%	34%	18%	12%	4%	20%	41%	26%	7%	2%	59%	20%	21%	59%
Mellau PD	49%	26%	28%	24%	15%	6%	3%	9%	24%	35%	22%	7%	n.a.	0-10%	n.a.	n.a.
Nenzing SD	29%	7%	34%	32%	26%	2%	6%	17%	33%	33%	9%	3%	34%	10%	50%	40%
Nenzing PD	50%	24%	30%	25%	16%	5%	3%	9%	24%	35%	22%	7%	n.a.	10-30%	n.a.	n.a.
All regions SD	34%	11%	27%	31%	24%	6%	6%	18%	36%	28%	8%	3%	26%	20%	15%	65%
All regions PD	51%	23%	28%	25%	17%	6%	7%	12%	28%	33%	14%	7%	n.a.	n.a.	n.a.	n.a.

SD = sample data; PD = population data; risk zone = flood return period of 300 years or less, or yellow/red risk zone; gender and age data: Statistics Austria (2014); household income data: Statistics Austria (2009); risk zone data: HORA (2015); percentages may not total 100 due to rounding.

1.2 Descriptive statistics of items

Table A.3: Descriptive statistics of items in trustee-specific factors

Factor	Item	Local government			Volunteers			Neighbors		
		N	Mean	SD	N	Mean	SD	N	Mean	SD
Trust	[Trustee] merits my full trust. (TRUST1)	1903	3.72	1.09	1905	4.45	0.67	1832	3.56	1.00
	[Trustee] acts in a fair way by his or her best intentions. (TRUST2)	1903	3.92	0.96	1901	4.45	0.66	1839	3.68	0.96
	[Trustee] is honest to me. (TRUST3)	1904	3.62	1.07	1901	4.28	0.74	1828	3.67	1.00
Competence	[Trustee] is able to judge the hazards correctly. (COMP1)	1907	3.76	1.03	1906	4.27	0.75	1831	3.32	1.01
	[Trustee] is able to protect me. (COMP2)	1902	3.44	1.11	1904	4.03	0.93	1831	3.01	1.14
Past performance	[Trustee] has acted in a competent way in the past. (PAST1)	1904	3.77	1.01	1893	4.43	0.65	1808	3.45	0.95
	[Trustee] has, so far, assessed the hazards correctly. (PAST2)	1899	3.69	1.03	1893	4.17	0.77	1817	3.30	0.98
Value similarity	[Trustee] has the same opinion as me. (VALUE1)	1887	3.81	0.98	1892	4.26	0.76	1900	4.03	0.88
	[Trustee] takes the hazards as seriously as I do. (VALUE2)	1908	4.04	0.98	1919	4.43	0.69	1903	4.05	0.92
	[Trustee] has the same values as me. (VALUE3)	1868	3.82	1.06	1874	4.24	0.79	1882	3.96	0.96

Items were introduced as: 'Regarding my threat from flooding, I believe that [trustee] …'; five-step response scale, 5=fully agree.

Table A.4: Descriptive statistics and factor loadings of items in cooperation factors

Factor	Item	Response scale	N	Mean	SD	Std. factor loading
Flood risk perception municipality	How likely do you consider a severe flood to be, within the next ten years in your municipality?	Ten-step, 10=very likely	1977	5.41	2.77	0.87
	What level of damage do you expect in your municipality, if a severe flood occurs?	Ten-step, 10=very high	1937	6.54	2.63	0.58
Flood risk perception own building	How likely do you consider a severe flood to be, within the next ten years at your own building?	Ten-step, 10=very likely	1934	4.22	2.76	0.95
	What level of damage do you expect at your own building, if a severe flood occurs?	Ten-step, 10=very high	1894	5.96	3.01	0.62
Flood risk perception fear	I am afraid of a potential flood.	Five-step, 5=fully agree	1951	3.17	1.33	0.82
	I am very worried about the potential threat of flooding.		1867	3.14	1.24	0.95
Risk perception climate change	I am sure that Austria is already affected by climate change.	Five-step, 5=fully agree	1909	4.16	0.97	0.61
	I am very worried how climate change will affect me.		1945	3.45	1.18	0.79
	I am very concerned about the impacts of climate change in Austria.		1867	3.52	1.13	0.92
Intention of implementing private measures	Purchase of private flood insurance	Six-step, 6=already implemented, 5=very likely, 2=very unlikely, 1=not feasible	1685	4.22	1.64	-
	No valuable belongings in the basement or on the ground floor		1641	3.54	1.77	-
	Emergency plan for all household members		1642	3.92	1.30	-
	Structural protection surrounding the building or at the building itself (e.g., dam, elevated building, waterproof walls and floors, heating installation on the upper floors)		1716	2.52	1.61	-
	Structural protection at parts of the building (e.g., waterproof doors and windows, electrical installations above water level, oil tank / fuel store securement, pressure flap on sewer pipe)		1693	2.79	1.50	-
	Coordination with neighbors (e.g., joint emergency plan, joint structural measures)		1694	3.00	1.37	-
	Provisional measures (e.g., sandbags, silicone sealing, flood barriers for windows and doors)		1709	3.55	1.37	-
	Mean index of all seven private measures		1803	3.36	0.98	-

Table A.4: Descriptive statistics and factor loadings of items in cooperation factors (cont.)

Factor	Item	Response scale	N	Mean	SD	Std. factor loading
Reliance on social support	In a flood event, I can count on support by others.	Five-step, 5=fully agree	1901	3.92	1.00	0.63
	Many people would help me during a flood.		1931	3.30	1.03	0.85
	In a flood event, many people would stand by me.		1860	3.35	1.01	0.88
Reliance on public protection	Thanks to public protection, I feel protected from a potential flood.	Five-step, 5=fully agree	1910	3.46	1.16	0.73
	I can entirely rely on public protection in my municipality.		1949	3.45	1.09	0.82
	Public flood protection makes me feel safe.		1945	3.54	1.12	0.81
	I trust in good flood protection in my municipality.		1868	3.76	1.04	0.80
Fatalism	A flood is just an uncontrollable act of nature.	Five-step, 5=fully agree	1909	3.90	1.15	0.67
	Ultimately, it is an act of fate whether someone is hit by a flood.		1930	2.99	1.26	0.59
	Besides all human causes, all floods are also an act of the powers that be.		1866	3.73	1.17	0.57
	Against the forces of nature, all efforts in flood protection are futile.		1866	2.47	1.14	0.54
Denial	Most people consider flooding a bigger problem than it actually is.	Five-step, 5=fully agree	1900	2.41	1.14	0.60
	Generally, the current flood hazard is being exaggerated.		1942	2.25	1.06	0.81
	The public discourse overstates the actual flood hazard.		1862	2.38	1.02	0.80
Wishful thinking	Sooner or later, the flood problem will solve itself.	Five-step, 5=fully agree	1900	1.59	0.90	0.42
	Sometimes I imagine that there will be no more flood hazards in the future.		1943	2.26	1.17	0.44
	The next flood will surely not occur as soon as currently expected.		1923	2.69	1.03	0.66
	The next flood will surely cause much less damage than is currently expected.		1852	2.64	1.06	0.69

1.3 Factor structure of trust, competence, past performance, and value similarity

Confirmatory factor analysis (CFA) establishes that the factors trust, competence, past performance, and value similarity are conceptually distinct between the three groups of trustees: local government, volunteers, and neighbors.

The four columns in Table A.5 correspond to separate CFAs for each of the four factors trust, competence, past performance, and value similarity. A comparison of trust, competence, past performance, and value similarity factors between trustees requires these factors to have the same conceptual meaning with respect to all trustees (Byrne *et al.* 1989; Steenkamp & Baumgartner 1998). To this end, we establish partial measurement invariance by restricting the unstandardized factor loadings of two items per factor to be equal across trustees (the respective items are indicated in Table A.5). The high factor loadings and acceptable model fit support that each of the four factors is distinctly attributed to the three trustees. RMSEA exceeds the .08 cut-off value in some models; however, the other fit indices yield satisfactory values.

Within each column-wise CFA, trustee assessments intercorrelate moderately. Table A.6 shows correlations in trust and value similarity. Competence assessments by trustee correlate with r=.49-.60. Intercorrelations by trustee within past performance amount to r=.37-.55. These intercorrelations suggest that the local government, volunteers and neighbors are not assessed independently by the citizens. This possibly reflects that regional disaster management is a joint, ongoing effort wherein the worldviews and abilities of various stakeholders are negotiated and partially aligned to each other. It is also likely that memberships of trusted groups overlap to a certain extent. A neighbor, for instance, may also work as a volunteer fire-fighter and thus belongs to both trusted groups. These medium-sized intercorrelations are not strong enough, though, to justify a trustee-overarching perspective. Comparisons to a restricted model assuming a single trustee-overarching factor yield substantially worse Chi^2 statistics (see bottom row in Table A.5). Thus, we may assume that the respondents do indeed differentiate between local government, volunteers and neighbors.

Within each trustee, however, the factors trust, competence, and past performance are not conceptually distinct. We find high collinearity between the three factors. This linear dependence prevents analysis of all factors in a joint CFA (i.e., row-wise CFAs in Table A.5 yield non-admissible solutions because of non-positive definite matrices, Schumacker & Lomax 2004). As discussed in

Section 2.2 of the paper, trust, competence and past performance suffer from conceptual overlap. Citizens seem to hardly differentiate between all dimensions of emotional (i.e., trust) and cognitive (competence) assessment or historical experiences (past performance) associated with a trustee.

Mean indices illustrate the substantial intercorrelations between trust, competence, and past performance within each trustee. Reliability of these indices is high (see Cronbach's Alpha in Table A.7). Intercorrelations between trust, competence, and past performance indices within each trustee exceed $r>.74$ (Table A.7). Value similarity, in contrast, shows good discriminant validity, since its intercorrelations with the indices of the other factors are substantially lower than between trust, competence and past performance.

Byrne, B., Shavelson, R. and Muthén, B. (1989) Testing for the equivalence of factor covariance and mean structures: The issue of partial measurement invariance. *Psychological Bulletin*, 105, 456-466.

Steenkamp, J. and Baumgartner, H. (1998) Assessing measurement invariance in cross-national consumer research. *Journal of Consumer Research*, 25, 78-90.

Table A.5: Confirmatory factor analyses on trust, competence, past performance, and value similarity

		**Trust**	Std. factor loading	**Compe-tence**	Std. factor loading	**Past perfor-mance**	Std. factor loading	**Value similarity**	Std. factor loading
Local government		TRUST1 #	.87	COMP1 #	.72	PAST1 #	.78	VALUE1 #	.86
		TRUST2	.88	COMP2 #	.99	PAST2 #	.96	VALUE2	.87
		TRUST3 #	.87	-	-	-	-	VALUE3 #	.89
Volunteers		TRUST1 #	.85	COMP1 #	.71	PAST1 #	.73	VALUE1 #	.84
		TRUST2	.86	COMP2 #	.90	PAST2 #	.83	VALUE2	.83
		TRUST3 #	.80	-	-	-	-	VALUE3 #	.90
Neighbors		TRUST1 #	.86	COMP1 #	.73	PAST1 #	.78	VALUE1 #	.90
		TRUST2	.92	COMP2 #	.95	PAST2 #	.93	VALUE2	.92
		TRUST3 #	.89	-	-	-	-	VALUE3 #	.92
Model fit	Chi² (df)		318 (26) **		284 (8) **		130 (8) **		670 (26) **
	CFI		.98		.94		.97		.95
	NFI		.97		.94		.97		.95
	RMSEA (10%-CI)		.075 (.068-.082)		.131 (.118-.144)		.087 (.074-.101)		.111 (.104-.119)
Model comparison to a single trustee-overarching factor Difference in Chi² (df)			5536 (1) **		1300 (1) **		1721 (1) **		6250 (1) **

* p<.05, ** p<.01.; # Trustee-invariant factor loading; CI=confidence interval.

Table A.6: Trustee-specific interrelations between value similarity and trust factors

	Value similarity Volunteers	Value similarity Neighbors	Trust Local government	Trust in Volunteers	Trust in Neighbors
Value similarity Local government	.57 **	.34 **	.75 **	-	-
Value similarity Volunteers	-	.51 **	-	.56 **	-
Value similarity Neighbors	-	-	-	-	.53 **
Trust in Local government	-	-	-	.48 **	.41 **
Trust in Volunteers	-	-	-	-	.43 **

* p<.05, ** p<.01; standardized path coefficients and correlations between latent factors; with respect to trust, intercorrelations refer to residual error terms of endogenous factors.

Table A.7: Intercorrelations between trust, competence, past performance, and value similarity indices

		Cronbach's Alpha	**Competence**	**Past performance**	**Value similarity**
Local government	Trust	.91	.87 **	.86 **	.69 **
	Competence	.84	-	.83 **	.65 **
	Past performance	.85	-	-	.64 **
	Value similarity	.90	-	-	-
Volunteers	Trust	.87	.78 **	.83 **	.46 **
	Competence	.75	-	.80 **	.44 **
	Past performance	.74	-	-	.46 **
	Value similarity	.89	-	-	-
Neighbors	Trust	.92	.74 **	.81 **	.49 **
	Competence	.82	-	.84 **	.41 **
	Past performance	.85	-	-	.46 **
	Value similarity	.94	-	-	-

* p<.05, ** p<.01; correlations between mean indices. Note that the correlations between value similarity and trust are smaller in size than in Table A.6 because item measurement error is higher for mean indices than for the factors in the structural equation model.

1.4 Mean comparisons in trust, competence, past performance, and value similarity

Table A.8: Mean levels of trust, competence, past performance, and value similarity indices by trustee, flood experience and risk zone

	Local government		Volunteers		Neighbors		F (df)
	Mean	SD	Mean	SD	Mean	SD	
Trust	3.75	0.95	4.39	0.62	3.64	0.91	693 (2/3678) **
Competence	3.59	0.99	4.14	0.76	3.16	1.00	945 (2/3652) **
Past performance	3.72	0.95	4.30	0.64	3.38	0.90	863 (2/3638) **
Value similarity	3.90	0.92	4.31	0.68	4.02	0.87	206 (2/3818) **

* p<.0125, ** p<.0025 (Bonferroni adjusted significance level to control for cumulative type 1 error, i.e., the significance level p is divided by the number of tests).

Table A.9: Mean levels of trust, competence, past performance, and value similarity indices by trustee and by flood experience

	Factor	No flood experience		Flood experience		t	df	p
		Mean	SD	Mean	SD			
Trust	Local government	3.84	0.88	3.50	1.12	6.09 #	719	**
	Volunteers	4.39	0.61	4.39	0.64	0.01	1886	
	Neighbors	3.61	0.88	3.69	0.99	-1.58 #	789	
Competence	Local government	3.69	0.93	3.33	1.12	6.33 #	752	**
	Volunteers	4.16	0.75	4.14	0.76	0.49	1888	
	Neighbors	3.15	0.98	3.20	1.05	-.90 #	844	
Past performance	Local government	3.84	0.86	3.44	1.13	7.10 #	711	**
	Volunteers	4.30	0.64	4.32	0.63	-.61	1876	
	Neighbors	3.33	0.86	3.51	0.99	-3.42 #	781	**
Value similarity	Local government	3.96	0.86	3.74	1.06	4.19 #	760	**
	Volunteers	4.30	0.68	4.35	0.66	-1.33	1905	
	Neighbors	3.96	0.87	4.19	0.83	-5.18	1897	**

* p<.001, ** p<.0002 (Bonferroni adjusted significance level to control for cumulative type 1 error). # corrected for inhomogeneous variances.

Table A.10: Mean levels of trust, competence, past performance, and value similarity indices by trustee and by risk zone

	Factor	Not in risk zone		In risk zone		Don't know		F	df	p
		Mean	SD	Mean	SD	Mean	SD			
Trust	Local government	4.09	0.96	3.61	1.04	3.73	0.90	20.62	2/1666	**
	Volunteers	4.60	0.53	4.38	0.64	4.35	0.63	16.13	2/1667	**
	Neighbors	3.90	0.94	3.66	0.89	3.56	0.91	13.85	2/1617	**
Competence	Local government	3.93	0.99	3.44	1.04	3.58	0.95	19.32	2/1663	**
	Volunteers	4.45	0.68	4.10	0.77	4.10	0.76	23.63	2/1671	**
	Neighbors	3.55	1.08	3.14	0.98	3.08	0.99	21.40	2/1612	**
Past performance	Local government	4.11	0.84	3.51	1.08	3.71	0.91	30.18	2/1663	**
	Volunteers	4.56	0.55	4.25	0.65	4.26	0.65	24.77	2/1659	**
	Neighbors	3.74	0.97	3.38	0.91	3.31	0.88	21.99	2/1601	**
Value similarity	Local government	4.09	0.88	3.88	1.02	3.85	0.89	7.16	2/1667	*
	Volunteers	4.41	0.69	4.39	0.64	4.27	0.69	7.33	2/1672	*
	Neighbors	4.11	0.86	4.12	0.84	3.95	0.86	6.88	2/1665	

* $p<.001$, ** $p<.0002$ (Bonferroni adjusted significance level to control for cumulative type 1 error).
